# Erythritol: An In-Depth Discussion of Its Potential to Be a Beneficial Dietary Component

**DOI:** 10.3390/nu15010204

**Published:** 2023-01-01

**Authors:** Tagreed A. Mazi, Kimber L. Stanhope

**Affiliations:** 1 Department of Community Health Sciences-Clinical Nutrition, College of Applied Medical Sciences, King Saud University, P.O. Box 10219, Riyadh 11433, Saudi Arabia; 2 Department of Nutrition, University of California Davis, 3135 Meyer Hall, One Shields Avenue, Davis, CA 95616, USA; 3 Department of Molecular Biosciences, School of Veterinary Medicine, University of California, Davis, CA 95616, USA

**Keywords:** erythritol, non-nutritive sweeteners, polyol, obesity, type II diabetes

## Abstract

The sugar alcohol erythritol is a relatively new food ingredient. It is naturally occurring in plants, however, produced commercially by fermentation. It is also produced endogenously via the pentose phosphate pathway (PPP). Consumers perceive erythritol as less healthy than sweeteners extracted from plants, including sucrose. This review evaluates that perspective by summarizing current literature regarding erythritol’s safety, production, metabolism, and health effects. Dietary erythritol is 30% less sweet than sucrose, but contains negligible energy. Because it is almost fully absorbed and excreted in urine, it is better tolerated than other sugar alcohols. Evidence shows erythritol has potential as a beneficial replacement for sugar in healthy and diabetic subjects as it exerts no effects on glucose or insulin and induces gut hormone secretions that modulate satiety to promote weight loss. Long-term rodent studies show erythritol consumption lowers body weight or adiposity. However, observational studies indicate positive association between plasma erythritol and obesity and cardiometabolic disease. It is unlikely that dietary erythritol is mediating these associations, rather they reflect dysregulated PPP due to impaired glycemia or glucose-rich diet. However, long-term clinical trials investigating the effects of chronic erythritol consumption on body weight and risk for metabolic diseases are needed. Current evidence suggests these studies will document beneficial effects of dietary erythritol compared to caloric sugars and allay consumer misperceptions.

## 1. Introduction

In 2015, the Manager of the Global Consumer Insights at General Mills reviewed the spectrum of consumers’ health views on sugars and sweeteners [1]. With the rankings starting with ‘good for health’ and descending toward ‘bad for health’, honey was at the top. Coconut sugar, agave, monk fruit and stevia were ranked in the top half. Sucrose, or granulated sugar, was near the center. On the bottom half of the rankings were sucralose, erythritol, xylitol, saccharin, aspartame, and high fructose corn syrup (HFCS). These rankings are in line with the results from the 2017 Canada Food Study online survey in which 1000 youth and young adults were asked to compare the healthiness of one of six sweeteners (aspartame, sucralose, stevia, agave, HFCS, “raw” sugar) to “table sugar” (or sucrose) [2]. The highest proportion of respondents rated HFCS, aspartame and sucralose as less healthy than sucrose, and the highest proportion of respondents rated stevia and “raw” sugar as healthier. Most respondents rated agave as either healthier or not different than sucrose. The results led the authors to conclude that consumers appear to base healthfulness perceptions on a sweetener’s level of “naturalness”.

There are two issues that consumers are disregarding when using ‘natural’ as their guide to the healthiness of sweeteners. First, the US. Food and Drug Administration (FDA) has not officially defined the term ‘natural’, though their longstanding policy suggests that a natural food must have nothing artificial or synthetic included or added to it [3]. Interestingly, the FDA has made it clear that because processing techniques can vary for the same sweetener, their determination as to whether the sweetener may be considered natural or not can also vary. The example is HFCS. In 2008 the FDA stated that the enzymatic transformation that turns corn syrup first into glucose and then turns some of the glucose into fructose disqualifies HFCS from being considered a natural sweetener [4]. However, a short time later, the FDA stated that HFCS could be considered natural when the enzymes causing these transformations are affixed to a column, thus not in contact or included in the final product [5]. It is likely that many or most consumers are unaware of such a distinction and assume all sweeteners labelled ‘natural’ are extracted intact from plants. However, under the current FDA policy regarding ‘natural’, biotechnological advancements that improve production, sustainability, and lower costs have already made this untrue, and make it increasingly unlikely it will be true in the future [6]. Even less defined than ‘natural’ is the word ‘raw’ in raw sugar; this marketing term implies that the sugar is minimally processed. In actuality, ‘raw’ or turbinado sugar can be up to 99.9% sucrose with negligible nutrient content from the residual molasses giving its brownish tint [7].

The second issue that consumers disregard is the scientific evidence indicating that being ‘natural’ does not ensure a sweetener is healthier than one that is not. Our recent publication comparing the consumption of sucrose-, HFCS- or aspartame-sweetened beverage (-SB) illustrates this [8]. Aspartame consists of two amino acids, aspartic acid and phenylalanine, that are naturally occurring in plant and animal proteins that humans consume. However, it takes several not naturally occurring chemical steps to bond them together via a methyl group and generate aspartame. Nevertheless, consumption of both sucrose- and HFCS-SB for 2 weeks induced detrimental and comparable changes in insulin sensitivity, liver and circulating lipids, lipoproteins, and uric acid profiles compared with aspartame-SB in young, healthy subjects [8]. Clearly, contrary to consumer perception, naturalness (i.e., extracted from a plant) does not relate to healthiness when comparing sucrose, HFCS, and aspartame.

It is likely the same misperception that leads consumers to rate sucralose and saccharin as less healthy than sucrose, since, like aspartame, they are synthesized chemically. However, it is less clear why erythritol, a noncaloric naturally occurring sugar alcohol, was perceived as ‘bad for health’ by consumers in the 2015 General Mills survey [1]. Similarly, in a more recent survey, 578 participants rated erythritol as the least preferred of the natural sweeteners and less preferred than sucralose [9]. Erythritol is likely one of the sweeteners with which consumer are least familiar, given its use in the US and European Union food supplies only dates to 2001 and 2003, respectively [10,11]. Furthermore, the 2019 global production would suggest most consumers have negligible exposure to erythritol. The 2019 global per capita production of the predominant caloric sweeteners, sucrose and HFCS, exceeds the erythritol production by over 3000-fold (67 gm/day for sucrose and HFCS; 0.023 gm/day for erythritol) [12,13]. However, erythritol’s production and consumer use are expected to increase [14]. If erythritol is used in all intended foods at maximum levels, the estimated future per capita intake is 32 gm/day (533 mg/kg) with a 90th percentile intake of 63.0 gm/day (1050 mg/kg) [15]. Therefore, the objective of this review is to summarize the scientific literature regarding the safety, production, metabolism, and health effects of erythritol and determine whether its ‘bad for health’ reputation is warranted.

## 2. Erythritol-Naturally Occurring and Endogenously Produced

Erythritol is approximately 70% as sweet as sucrose and has a mild cooling effect in the mouth with no aftertaste [16]. Erythritol is a naturally occurring sugar alcohol (or polyol) that is found a variety of fruits such as melon, watermelon, pears, grapes; and in fermented foods such as cheese, soy sauce [17,18,19]. Erythritol is also detected in plasma and urine in human subjects and animals [20,21]. It was detected in the plasma and urine of a child with an inborn error of the pentose phosphate pathway (PPP) [21]. Later Hootman et al. demonstrated that erythritol is endogenously produced in healthy human erythrocytes from glucose via PPP [22]. The PPP is a branch of glucose metabolism, present in all organisms, that synthesizes building block for nucleic acid and DNA; generates nicotinamide adenine dinucleotide phosphate hydrogen (NADPH), which is an essential co-factor in many anabolic reactions such as synthesis of fatty acids and non-essential amino acids; and regenerates the antioxidant, glutathione [23]. Schlicker et al. confirmed production of erythritol via PPP in human lung cancer cells and characterized two NADPH-dependent enzymes that catalyze the reduction of erythrose to erythritol, alcohol dehydrogenase 1 (ADH1) and sorbitol dehydrogenase (SORD) [24]. As these enzymes are highly expressed in liver and kidney, the authors [24] further proposed these metabolically active tissues as main contributors to endogenous synthesis of erythritol in mammals. However, factors influencing endogenous erythritol production require investigation.

## 3. Commercial Production of Erythritol

Erythritol occurs in fruits at levels too low to allow it to be extracted economically. It can be produced chemically, however, this method is also not cost-efficient [25]. In the 1950s, traces of erythritol were found in residue of blackstrap molasses fermented by yeast [26]. This led to the discovery that erythritol can be produced via fermentation by yeast and yeast-like fungi via PPP [25]. Erythritol can also be produced by some lactic acid bacteria from glucose via the phosphoketolase pathway [27]. Fermentation by yeast and yeast-like fungi is currently used as a cost-effective method for commercial large-scale erythritol production utilizing substrates such as glucose, fructose, xylose, sucrose, cellulose, and glycerol [25,27]. Following fermentation, the fermented broth is heated, filtered to remove microorganism and other impurities before it is dried into crystals. The erythritol yields have been increased by optimizing the fermentation parameters and/or by gene-targeting biotechnologies to produce strains with higher activity of enzymes involved in synthesis pathways and/or lower activity of enzymes that enable the organism to utilize erythritol. Methods directed towards improving the cost-efficiency and bio-sustainability of production continue to be investigated, including utilizing readily available byproducts such as molasses or employing bacteria capable of generating the erythritol from wheat straw [26].

Because erythritol occurs in nature, the FDA considers microbial-produced erythritol to be a natural sweetener [3]. Possibly many consumers do not agree and this explains the low health perception ratings that erythritol received in the surveys already discussed [1,9]. However, preliminary results of a 2020 survey showed that 77% of 278 respondents had a positive or very positive attitude towards microbial applications in food production [28]. Interestingly, consumers who considered themselves “environmentally concerned” were more positive towards microbial applications in food compared to those who considered themselves “health concerned”. This perspective of the latter consumers may change, however, as the potential for reprogramed microbes to meet the planet’s increasing demands for the production of environmentally friendly biomolecules related to nutrition, pharmaceuticals, and even biodegradable plastic, continues to grow [6,29].

## 4. Erythritol Safety

Safety reviews for erythritol were conducted by multiple regulatory entities. In 2000, The Joint Expert Commission on Food Additives of the World Health Organization and the Food and Agriculture Organization (JECFA) established the Acceptable Daily Intake (ADI) for erythritol as “not specified” [30]. In 2001, the FDA classified erythritol as “generally recognized as safe” (GRAS) substance for use by the general population as a sweetener and flavor-enhancer in food and beverage [10]. In the following years, the FDA approved other GRAS notices for erythritol and erythritol ingredients for uses including as non-nutritive sweetener, flavor enhancer, stabilizer, and thickener in a variety of foods such as bakery fillings, cakes and cookies, frozen dairy desserts, puddings, yogurt, chewing gum, candies, and reduced and low-calorie beverages [15,31,32,33]. In the European Union, The European Food Safety Authority (EFSA) approved erythritol in 2003 as safe for use as a non-sweetening food additive [11]. Later, in 2006, it was permitted as sweetener for use in all food applications as with other polyols [34]. The EFSA authorized erythritol use in beverages as non-sweetening additive in 2010 [35], and as sweetener in 2015 [36].

The safety of erythritol is based on extensive evidence from animal and human studies on its absorption, distribution, metabolism, and excretion; along with short and long-term toxicological studies to examine potential reproductive, developmental and genotoxicity; as well as any mutagenic and carcinogenic effect, as reviewed in detail [37]. In one long-term rat study (104–107 weeks) that examined the toxicity and carcinogenicity of diets containing 0, 2, 5, or 10% erythritol (equivalent to 1.0, 2.6, and 5.4 gm/kg/day in females and 0.9, 2.2, and 4.6 gm/kg/day in males, respectively), erythritol consumption did not affect the survival of the animals, and showed no signs of nephrotoxicity, tumor-inducing or tumor-promoting changes [38].

In general, excessive intake is of sugar alcohols is associated with undesirable gastrointestinal effects, including nausea, abdominal bloating, and diarrhea. These side effects are attributed to the fact that sugar alcohols are poorly absorbed, thus they induce an osmotic effect and water retention in the intestine [25]. In addition, unabsorbed polyols can undergo fermentation by intestinal microbiota resulting in gas formation. However, most of an erythritol load is absorbed with relatively minimal amount reaching the colon [37]. Consequently, erythritol is better tolerated and is associated with less gastrointestinal side effects than sorbitol and xylitol at comparable doses [37]. The tolerance upper limits for erythritol are higher than for other polyols (0.66 gm/kg/day in men and 0.80 gm/kg/day in women) [39]. However, larger doses (1 gm/kg/day) are reported to be well-tolerated [40].

## 5. The Metabolism of Erythritol

Consisting of four carbons, erythritol is smaller and with lower molecular weight compared to the other commonly consumed sugar alcohols: xylitol (five carbons), sorbitol and mannitol (six carbons). The sugar alcohols are absorbed from the small intestine by passive diffusion in a size-dependent manner. Thus, erythritol is absorbed into the blood at a higher and faster rate than the larger sugar alcohols. Once in the blood, a major proportion of erythritol is un-metabolized and excreted unchanged in the urine. Studies in humans have shown that approximately 90% of a 20 gm-dose is recovered in the urine within 24 h, whereas 80% is recovered within 24 h when a 1 gm/kg dose is utilized [37]. The fate of the 10–20% erythritol not recovered in the urine is not clear. Hootman et al. have suggested that 5–10% of erythritol in blood may be oxidized to erythrose and further to erythronate [22].

It has mainly been assumed that the relatively small amount of unabsorbed erythritol passes to the colon. Data from 24 h in vitro studies utilizing human fecal samples indicate no evidence of erythritol being metabolized by gut microbiota [41]. This corroborated earlier evidence from humans consuming radio-labelled erythritol (25 gm). The study demonstrated that erythritol was almost completely recovered in urine, radiolabeled CO_2_ was not detected, and H_2_ gas did not increase in breath samples collected for 6 h after dosing [42]. This indicated that the nearly all of the absorbed erythritol was not metabolized systemically and the unabsorbed portion that transitioned to large intestine was not metabolized by gut microbiota.

Evidences from animals are not in agreement with data from human studies, and indicate 6–10% of consumed erythritol is metabolized by colonic microbiota with only about 1% excreted as erythritol in feces [37]. Consistent with erythritol fermentation by intestinal microbiota, mice that were fed a high-fat diet and administered water containing 5% erythritol had increased levels of short-chain fatty acids in their serum and feces [43]. Animal studies also suggest that the rates of erythritol metabolism by microbiota are higher in animals that have been pre-adapted to high erythritol diets [37]. As recently reviewed, the long-term in vivo studies that are needed to understand the metabolism of erythritol by human gut microbiota, as well as the effects of erythritol on human gut microbiota, have not been conducted [44]. Despite the lack of certainty regarding the metabolic processing of erythritol in the colon, and due to the uncertain fate of 10% of a 0.3 gm/kg dose, erythritol’s nutritive value has been estimated to be less than 0.4 kcal/gm [45]. However, for the purposes of nutritional labelling, erythritol it is assumed to contain 0 kcal/gm as compared to 2.4 kcal/gm for the other sugar alcohols [46].

## 6. Health Effect of Erythritol

### 6.1. Effects of Erythritol on Tooth Decay

The first studies reporting that sorbitol or xylitol have positive effects on dental health are more than 50 years old [47,48]. The most recent evidence specific to erythritol has been extensively reviewed by de Cock et al. [49]. Three clinical studies lasting 2–6 months, and one lasting 3 years [50], have demonstrated that consumption of candy or chewable tablets containing erythritol (5–7.5 gm/day) inhibited formation of dental plaque in young children, teenagers, and adults [51,52,53]. In vitro studies suggest that erythritol, and also sorbitol and xylitol, inhibit bacterial growth due to an osmotic effect [49]. However, erythritol causes more marked inhibition of bacterial growth than sorbitol and xylitol [51], likely due to its ability to passively pass through bacterial cell membrane where it interferes with growth pathways [54]. The ability of erythritol compared with sorbitol or xylitol to slow the development of dental caries was demonstrated in two 3-year studies in children, 8–9 years old [55] and 14–15 years old [56]. De Cock et al. concluded that the evidence demonstrates that erythritol is more effective than sorbitol or xylitol at maintaining and improving oral health [49].

### 6.2. Effects of Erythritol on Glycemia and Insulin Secretion

Studies in human subjects; lean and obese, with diabetes and without; have clearly demonstrated that acute doses of erythritol (20–75 gm) do not affect blood levels of glucose or insulin [45,57,58]. Livesey utilized the available data to calculate the glycemic and insulinemic indices of the sugar alcohols [59]. Compared to the glucose reference score of 100 for both indices, the glycemic index for erythritol was 0, and the insulinemic index was 2. While these characteristics make erythritol potentially beneficial for subjects with diabetes, controlled clinical trials examining the effect of erythritol intake in subjects with diabetes are limited, with only one published study to support this notion. In a 2-week intervention trial, patients with diabetes consumed erythritol (20 gm/day) and exhibited a significant decrease in hemoglobin A1c (HbA1c) from 8.5 to 7.5% [58]. In acute clinical studies (24 h or less), erythritol delayed gastric emptying and glucose absorption from the small intestine, which was accompanied by dose-dependent increases in gut hormones: glucagon-like peptide 1 (GLP-1), cholecystokinin (CCK), and Peptide YY (PYY) [57,60].

In multiple diabetic animal models, erythritol treatment reduces blood glucose [61,62]. Compared to an oral glucose bolus, the acute feeding of erythritol with a glucose bolus delayed gastric emptying, attenuated the rise in blood glucose, improved oral glucose tolerance test (OGTT) response, increased expression of muscle glucose transporter type 4 (GLUT4, required for transporting glucose into cells), and increased expression of insulin receptor substrate 1 (IRS-1, required for insulin function) [63]. Studies by Wen et al. provided a mechanism by which erythritol may reduce or delay glucose absorption from the small intestine [61]. First, they reported that the administration of bolus starch with erythritol to diabetic mice resulted in reduced postprandial glucose levels via inhibited α-glucosidase activity, an intestinal epithelial enzyme that catalyzes the hydrolysis of glucose polymers into glucose. Then, using a computational molecular modeling technique, they demonstrated that erythritol can directly interact with α-glucosidase by competitively occupying the active catalytic pocket.

A recent clinical dietary intervention study does not support the above findings. Bordier et al. compared the effects of chronic intake (5–7 weeks) of erythritol (12 g 3 times daily), xylitol (8 g 3 times daily) or no sweetener on intestinal glucose absorption in obese subjects [64]. OGTTs were conducted pre- and post-intervention utilizing 3-Ortho-methyl-glucose (a marker of glucose absorption). The results showed that chronic consumption of erythritol, or xylitol, had no between- or within-group effects on intestinal glucose absorption. However, it is important to note that in the animal studies [61,63], the glucose or starch boluses were preceded by or paired with erythritol consumption, while in the clinical study the OGTTs were performed after an overnight fast and without concurrent erythritol consumption [64]. If the proposed model, that erythritol can directly interact with α-glucosidase by competitively occupying the active catalytic pocket [61], is valid, then it is reasonable that erythritol can only impede intestinal glucose absorption when glucose and erythritol are consumed in proximity.

### 6.3. Effects of Erythritol on Energy Intake and Body Weight

Surprisingly, there appears to be only two clinical trials in which the effects of erythritol consumption on body weight were reported. One of these trials lasted only seven days, and the body weight of 12 healthy men consuming erythritol (1 gm/kg/day) was not affected [40]. In a two-week trial, body weight was monitored in 7 patients with type II diabetes who consumed erythritol (20 gm/day) [58]. While mean body weight decreased by 2 kg over the two weeks, the change was driven by marked weight loss in three of the subjects and was not significant. Acute trials in humans have shown that erythritol administration stimulated the secretion of gut hormones modulating satiety and energy intake; GLP-1, CCK and PYY; compared to water or equal amounts of glucose [57,60]. In an acute 3-way crossover study, consumption of an isocaloric, but not an iso-volumetric, erythritol-sweetened custard increased GLP-1 and PYY compared with a sucrose-sweetened custard, and these increases were associated with a reported reduction of hunger in lean subjects [65]. However, the energy consumption during the follow-up ad libitum feeding trials was not affected by the erythritol compared to sucrose preloads. In another acute crossover trial, Sorrentino et al. found that consumption of erythritol-SB suppressed the appetite hormone ghrelin and increased self-reported satiety compared to a comparably sweetened aspartame beverage [66]. The authors suggest that the effects of erythritol on the satiety/appetite hormones are due to it having a higher osmolality than iso-caloric or iso-sweet sucrose, or other non-nutritive sweeteners [66]. There are data demonstrating that, regardless of caloric content, increased osmolarity in the duodenum induces alterations in satiety/appetite hormones and reduces hunger [67,68].

Several recent long-term mice studies investigating the effects of erythritol consumption on body weight and satiety hormones have been conducted, but the findings are not consistent with regard to body weight, adiposity, and energy expenditure. In a 12-week intervention, mice consuming water containing 5% erythritol along with a high fat diet exhibited comparable food intake, but less weight gain and higher energy expenditure than the group consuming high fat diet with water (*n* = 6/group) [43]. Similarly, mice consuming a high fat diet that contained 5% (*w*/*w*) erythritol for 16 weeks had lower body weight and higher muscle mass than the control group on a pair fed high fat diet [69]. The same research group reported that diabetic mice consuming a normal fat diet supplemented with 5% erythritol for 16 weeks accumulated less white adipose tissue than the control group on the normal fat diet (*n* = 12/group) [70]. However, Ortiz et al. reported that four separate studies showed that 8- or 20-week-old mice consuming low or high fat diets supplemented with 4% erythritol (*w*/*w*) for 8 weeks exhibited gains in body weight and adiposity that were comparable to their respective control groups (*n* = 8/group) [20]. Even more contradictive, Mitsutomi et al. reported that mice that consumed high fat diet with 4% erythritol in water for 4 weeks accumulated white adipose tissue and decreased dark phase oxygen consumption compared to the control group (*n* = 5/group) [71]. However, in the 2-year safety study cited above [38], male rats consuming the 5 and 10% erythritol diets gained less body weight than male rats consuming the un-supplemented diet (*n* = 90/group). Lower body weights were apparent at 3 weeks in the rats consuming the 10% diet and at 8 weeks in the rats consuming the 5% diet [38].

In summary, while erythritol is an iso-volumetric and non-nutritive replacement for added sugar that may also promote satiety hormone secretion, clinical studies investigating its effects on body weight and adiposity are lacking. Data from animal studies are not consistent, however the longer studies (≥12 weeks) demonstrate positive effects of erythritol consumption on body weight or composition [38,43,69,70] and the shorter studies (4 & 8 weeks) do not [20,71]. More studies, especially clinical trials, are clearly needed to determine the effects of chronic erythritol consumption on body weight, energy intake and expenditure, and satiety/appetite hormones.

### 6.4. Effects of Erythritol on Risk Factors for Cardiometabolic Diseases

Given the lack of clinical dietary intervention studies that have investigated effects of chronic erythritol consumption on body weight, it is to be expected that clinical dietary intervention studies that have investigated effects of erythritol consumption on risk factors for cardiometabolic diseases are also lacking. An exception is a promising pilot study conducted in 24 patients with type II diabetes who consumed 26 gm/day of erythritol for 4 weeks. These patients exhibited reduced arterial stiffness and improved endothelial function [72]. Endothelial dysfunction contributes to the pathogenesis of cardiovascular disease in type II diabetes and predicts cardiovascular events [73]. In a sub-group of 12 subjects with systolic blood pressure > 130 mmHg, these changes were associated with reduced systolic blood pressure and central pulse pressure [72]. These beneficial effects of erythritol on endothelial function are supported by rodent studies [74] and in vitro studies [75], however they need to be confirmed in clinical trials that include appropriate controls. The pilot clinical trial did not include a control group [72].

The effects of sustained consumption of erythritol on other outcomes associated with cardiometabolic disease; such as lipid and lipoproteins, uric acid, glucose tolerance and insulin sensitivity, liver triglyceride content and enzymes, and inflammatory factors; have not been studied in humans. Some of these risk factors have been studied in the previously cited dietary intervention studies conducted in mice and the changes paralleled the effects on body weight. The 12-week intervention, which documented lower weight gain and higher energy expenditure in the mice consuming water containing 5% erythritol along with a high fat diet, also showed lowered inflammation and liver fat accumulation, and improved glucose tolerance [43]. Diabetic mice consuming a normal diet supplemented with 5% erythritol for 16 weeks exhibited lowered plasma levels of interleukin-6 and resistin along with less white adipose tissue [70]. The four separate studies that showed 8 weeks of erythritol supplementation did not affect body weight or adiposity also showed no beneficial or adverse effects on glucose tolerance [20]. In stark contrast to the 12-week study [43], the 4-week study, which showed increased white adipose tissue and decreased oxygen consumption after erythritol supplementation, also showed increased liver and muscle fat and glucose intolerance [71].

The lack of human studies investigating the effects of erythritol on risk factors for cardiometabolic disease, and the inconsistent results from animal studies, prevent a clear understanding of the potential for erythritol to serve as a healthy replacement for added sugar in the diet of healthy people and patients with type II diabetes. This understanding is further clouded by the results from observational studies.

### 6.5. Circulating Erythritol as a Candidate Predictor of Metabolic Risks

Paradoxically, several metabolomic profiling studies reported a positive association of circulating erythritol with impaired fasting glucose and type II diabetes-related vascular complications [76,77,78]. Additionally, a higher serum or plasma erythritol was found to predict incidence of central adiposity gain (over 9 months), type II diabetes (over 20 years), and coronary heart disease (CHD) (over 30 years) [22,79,80]. While these association may seem contradictory to the aforementioned favorable metabolic effects, it is important to note that none of these studies evaluated diet. Furthermore, in the two largest studies that showed these associations [79,80], sample collection occurred in the U.S. before erythritol was approved as a dietary component. Obviously, dietary erythritol intake does not explain the associations between circulating erythritol and cardiometabolic disease that were observed in these two studies. However, even for studies in which samples were collected after erythritol introduction into the food supply, it needs to be considered whether the global per capita production of erythritol, estimated at 0.023 g/day in 2019 [12,13], supports consumption levels high enough to obtain discernable associations with cardiometabolic disease. It seems unlikely.

On the other hand, there is evidence to suggest that it is plausible that endogenous production of erythritol associates with cardiometabolic disease [81]. As previously stated, Hootman et al. demonstrated that erythritol is endogenously produced in healthy human erythrocytes via the PPP [22]. The PPP runs parallel to glycolysis, and many glycolytic/gluconeogenetic intermediate metabolites feed into it. Therefore, a diet rich in glucose and/or fructose, which are consumed at much higher levels than erythritol, can likely influence endogenous erythritol production, and this awaits investigation. Additionally, there is growing evidence for a role of PPP in modulating insulin sensitivity and obesity-induced inflammation in multiple tissues such as adipocytes, hepatocytes, skeletal muscles, and pancreatic β-cells [82,83]. Moreover, animal studies indicate a bi-directional link between hyperglycemia and the overexpression of glucose-6-phosphate dehydrogenase (G6PD), the first and rate limiting enzyme in PPP observed in adipocytes and hepatocytes [82]. In subjects with obesity, insulin resistance and diabetes, the PPP is reported to be dysregulated [83,84]. Therefore, it would be reasonable to postulate that hyper- or impaired glycemia underlies the elevated circulating erythritol observed in the observational studies [22,76,77,78]. This is supported by findings from a large prospective study by Rebholz et al. [79], which showed an association of erythritol with incident of type II diabetes. However, when fasting glucose was added to the statistical model, this association was attenuated. Menni et al. [76] showed an elevated plasma erythritol in subjects with impaired fasting glucose (IFG, 5.6 mmol/L < fasting glucose < 7 mmol/L) compared to the control group (3.9 mmol/L < fasting glucose < 5 mmol/L). Similarly, as hyperglycemia is implicated in the development and progression of diabetes-related vascular complications [85,86], findings from Shao et al. and Chen et al. [77,78] in diabetic nephropathy and diabetic retinopathy, also support an underlying hyperglycemia with an elevated erythritol. Thus, the higher circulating erythritol levels, reported to be associated with cardiometabolic diseases, is likely attributed to endogenous production via up-regulated PPP, with implications for hyper- or impaired glycemia. However, whether elevated erythritol contributes to the pathogenesis of cardiometabolic diseases or is a benign marker of PPP dysregulation, remains to be elucidated. In mice, high levels of plasma erythritol appear to be benign, at least over a period of 8 weeks. Ortiz et al. measured fasting plasma erythritol in mice before and after consumption of low or high fat diets supplement with or without 4% erythritol [20]. By the end of the 8-week intervention, fasting plasma erythritol levels in the erythritol-fed mice were 20–60 times higher than levels in the control mice. However, there were no differences in body weight, adiposity, or glucose tolerance between the groups. It will take studies longer than 8 weeks to ensure that increased plasma erythritol levels induced by dietary erythritol are benign. However, it is worth re-stating that the safety study in which rats consumed a 10% erythritol diet for 2 years revealed positive effects on body weight, and no adverse effects on numerous biochemical or histopathological parameters, including alanine aminotransferase, aspartate aminotransferase, and gamma γ-glutamyltransferase [38]. These liver enzymes increase in liver disease and are associated with the risk of type 2 diabetes, metabolic syndrome, and cardiovascular disease [87,88,89].

## 7. Conclusions

Erythritol is a naturally occurring, safe, and non-nutritive sugar alcohol. Compared to other sugar alcohols, evidence from human studies indicate that it is mostly absorbed and excreted in urine unmetabolized. Minimal amounts reach the colon; therefore, it is better tolerated, with less undesirable gastrointestinal effects. Consumption of erythritol does not increase circulating glucose or insulin and acute clinical trials suggest it promotes gut hormone release. The scientific evidence demonstrating that consumption of erythritol has beneficial effects on oral health is strong, with long-term controlled clinical trials conducted in both children and adults. In contrast, the long-term controlled clinical trials supporting erythritol as a beneficial dietary component that can lower glucose levels, body weight and risk factors in patients with type II diabetes, obesity or metabolic syndrome are almost completely lacking. The exceptions are two published trials that provide evidence that consumption of erythritol lowered HbA1c [58] and improved endothelial function [72] in patients with type II diabetes. These results are supported by rodent studies and in vitro studies, but need to be confirmed in long-term randomized controlled trials that also investigate the effects of erythritol consumption on body weight, insulin sensitivity, and risk factors for cardiometabolic disease. It is also important to elucidate the positive relationship between circulating erythritol and cardiometabolic diseases observed in epidemiological studies. The plausible explanation is that plasma erythritol is benign biomarker of PPP dysregulation resulting from glucose or fructose-rich diets or conditions that increase or impair glycemia, but this needs to be confirmed in clinical trials.

The studies summarized in this report suggest that future trials have the potential to document beneficial effects of erythritol on human health. If such results are generated, this will be impactful dietary information for patients with type II diabetes, obesity and metabolic syndrome, and their physician and dietitians. It is possible, however, that future trials may reveal no beneficial effects of erythritol on health outcomes. Yet, it is important to understand, null effects of erythritol are positive results when compared with the well-documented detrimental effects of sucrose or HFCS consumption [8,90,91,92,93,94,95,96,97]. All consumers have the potential to be benefitted when their perception that sucrose is a healthier dietary sweetener than erythritol is corrected with scientific evidence.
